# Telomerase-Dependent and Independent Telomere Maintenance and its Clinical Implications in Medullary Thyroid Carcinoma

**DOI:** 10.1210/jc.2014-1158

**Published:** 2014-04-23

**Authors:** Na Wang, Dawei Xu, Anastasios Sofiadis, Anders Höög, Vladana Vukojević, Martin Bäckdahl, Jan Zedenius, Catharina Larsson

**Affiliations:** Department of Oncology-Pathology (N.W., A.S., A.H., C.L.), Department of Medicine, Division of Hematology (D.X.), Department of Clinical Neuroscience, Center for Molecular Medicine (V.V.), Department of Molecular Medicine and Surgery (M.B., J.Z.), Karolinska Institutet, SE-171 76 Stockholm, Sweden; Cancer Center Karolinska (N.W., A.S., A.H., C.L.), Department of Pathology-Cytology (A.H.), Department of Breast and Endocrine Surgery (M.B., J.Z.), Karolinska University Hospital, SE-171 76 Stockholm, Sweden

## Abstract

**Context:**

Telomere maintenance via telomerase activation and the alternative lengthening of telomeres (ALT) mechanism was assessed in medullary thyroid carcinoma.

**Setting and Design:**

In total, 42 medullary thyroid carcinomas (MTC) were studied including 24 rearranged during transfection (*RET*)- mutated cases. Relative telomerase reverse transcriptase (*TERT*) expression, splice forms, and telomere length were determined by PCR-based methods, and telomerase activity by ELISA. The ALT mechanism was detected by Southern blot analysis and immunofluorescence.

**Results:**

*TERT* expression and telomerase activity were detected in 21/42 tumors (50%), and was independent of the common somatic M918T *RET* mutation. Mean telomere length was shorter in MTCs compared with thyroids. Telomerase activation was associated with large tumor size (*P* = .027), advanced clinical stage (*P* = .0001), and short survival (*P* = .0001). Full-length *TERT* and the α^−^ and β^−^-deletion forms were revealed, and the full-length form was associated with short survival (*P* = .04). A subset of cases without telomerase activation showed involvement of the ALT mechanism, which was associated with a low MIB-1 proliferation index (P = .024).

**Conclusions:**

Stabilization of telomeres by telomerase activation occurs in half of the MTCs and by the ALT mechanism in a subset of cases. Telomerase activation may be used as an additional prognostic marker in medullary thyroid carcinoma.

Telomeres shorten with each cell division in most somatic cells. When the telomere length is reduced below a critical value, the Hayflick limit is approached and cellular senescence is triggered (1, 2). Therefore, immortal cells and cancer cells must recruit a mechanism for telomere stabilization to prevent senescence. Indeed, activation of telomerase in the presence of short telomeres is one of the most common features in many human cancers (3, 4). Telomerase is a ribonucleoprotein with two key subunits, the telomerase reverse transcriptase and the RNA component. It elongates telomeric DNA in human cells and telomerase activity is detectable in 85–90% of human malignancies. Telomerase activation is therefore a characteristic feature and a potential therapeutic target for cancer treatment (5). An alternative lengthening of telomeres (ALT) mechanism has also been described, which is thought to be based on homologous recombination (6, 7).

Medullary thyroid carcinomas (MTC), arising from calcitonin-producing parafollicular cells (C cells), account for 5–8% of human thyroid cancers (8). Approximately 75% of the cases are sporadic and 25% present as multiple endocrine neoplasia (MEN) type 2 (MEN 2) including syndromic MEN 2A and MEN 2B or familial MTC (9). The development of MTC is strongly linked to activating mutations of the *RET* (Rearranged during Transfection) proto-oncogene. Almost all MEN 2 cases carry a constitutional *RET* mutation, and approximately 40% of sporadic MTCs have a somatic *RET* mutation M918T with prognostic implications (9, 10).

Although the involvement of *TERT* and telomerase has been described in follicular cell-derived thyroid cancer, the extent and role in MTC is less studied. Recently, activating *TERT* promoter mutations were identified as a cause of telomerase activation and associated with poor prognosis in follicular, papillary, and anaplastic thyroid carcinomas; however, such mutations have not been observed in MTC (11–15). Alternative splicing of *TERT* has been reported in follicular cell-derived thyroid tumors (16). Several *TERT* transcripts have been found, including three deletions and four insertions, which may affect telomerase enzyme activity and biological functions (17–19). Four insertions and the β^−^ and γ^−^ deletion result in nonfunctional proteins whereas the α^−^ deletion is a dominant negative inhibitor of telomerase activity (20, 21). In follicular and papillary thyroid carcinomas three transcripts were detected including full-length, α^−^ deletion and β^−^ deletion (16).

To further elucidate telomere stabilization in MTC we characterized a panel of tumors for activation of telomerase or the ALT mechanism in relation to *RET* mutational status, clinical characteristics, and patient outcomes.

## Materials and Methods

### Patients and tissue specimens

The study includes all patients operated on for MTC between 1986 and 2010 in the Karolinska University Hospital, Stockholm, from whom a fresh frozen tissue sample was available. All 42 cases were operated without preceding or subsequent chemotherapy or irradiation therapy. The details concerning age, sex, tumor size, TNM classification, MIB-1 proliferation index, *RET* mutation status, follow-up, and clinical outcome are summarized in Supplemental Table 1. Patients were followed up regularly (at 3, 6, and 12 months postoperatively, thereafter every 6 months for 5 years and then yearly), with clinical examination and measurement of basal serum calcitonin levels and radiology when suggested. In addition, 24 histopathologically verified noncancerous thyroid tissue samples obtained from patients surgically treated for other thyroid tumors than MTC were included as references. All tissue specimens were obtained through the Karolinska University Hospital Biobank. Paraffin-embedded tissues were also obtained for immunohistochemical purposes from a subset of MTCs and normal thyroid tissues. Histopathological classification of specimens was performed according to the criteria of the World Health Orginization Committee (8). *RET* mutation status was based on sequencing of exons 10, 11, 15, and 16 of *RET* in all MTC tissues (Wang et al, in preparation). Data for proliferation analysis determined by Ki-67 immunohistochemistry using the MIB-1 antibody was available for 23 of the cases (for cases with multiple surgical samples the highest MIB-1 index was chosen as representative). Informed consent was obtained from all patients and the study of the tissue samples was approved by the local Ethics Committee.

### Quantitative real-time PCR

Total *TERT* expression was quantified by Quantitative real-time PCR (qRT-PCR) using Taqman Gene Expression Assays (Applied Biosystems) for *TERT* (Hs00 972656_m1) and *18S* rRNA (Hs99999901_s1). *TERT* splice variants were analyzed using the methodology from Wang et al (16). The experimental procedures and quantifications are described in the Supplemental Material.

### Assessment of telomerase activity

Telomerase activity was assayed in protein extracts with TeloTAGGG Telomerase PCR ELISA kit (Roche Diagnostics GmbH) based on the telomeric repeat amplification protocol described by Kim and Wu (22). Lysis buffer and HEK-293 cells served as negative and positive controls. All samples were performed in triplicate. The level of telomerase activity was determined in arbitrary units based on absorbance at OD_450_-OD_690_.

### Assessment of telomere length

Mean relative telomere lengths were determined in MTCs and thyroid samples by real-time PCR according to published methodology (23), and telomere lengths were assessed by Southern blot analysis. The analyses are further described in the Supplemental Material.

### Detection of ALT-associated promyelocytic leukemia bodies (APBs)

APBs were detected by combined telomere fluorescence in situ hybridization (Tel-FISH) and promyelocytic leukemia (PML) immunofluorescence as described (24). Representative tissue sections from 19 MTCs (nine telomerase negative and ten telomerase positive), and five normal thyroids were included. Telomerase-positive HeLa cell line was used as negative control. A detailed description of the methodology and scoring is given in the Supplemental Material.

### Statistical analysis

The statistical analyses were performed using SPSS software version 18.0 for Windows. Differences between groups were evaluated by χ^2^ test or Fisher's exact test (where appropriate) and Mann-Whitney *U* test. Spearman rank order correlation was performed to analyze correlation between telomerase expression and telomerase activity as well as MIB-1 proliferation index and telomerase activity. Multivariate logistic regression was used to calculate the odds ratios and their 95% confidence interval (CI). Survival curves were illustrated by Kaplan-Meier plots, and significance was calculated by log-rank test. *P* values < .05 were regarded as statistically significant.

## Results

### Clinical and genetic characterization of the study cohort

The clinical details for the 39 sporadic MTC and three MEN 2 cases are summarized in Supplemental Table 1. Of the 39 sporadic patients, 25 had a poor outcome at the end of follow-up, of which 12 died of the disease and 13 were alive with persistent disease, 12 were alive and free of disease at the end of follow-up, and 2 died resulting of causes unrelated to MTC. The three MEN 2 cases included a man with familial MTC diagnosed at age 14 years who survived for more than 34 years and two women with MEN 2A diagnosed at 28 and 54 years of age who are still alive after 22 and 9 years, respectively. In total, 24 of the 42 cases (57%) were *RET*-mutation positive (Supplemental Table 1). All three cases with familial disease harbored a constitutional mutation that was also present in the corresponding tumor DNA. Among the 39 sporadic MTCs, 17 tumors exhibited the common mutation M918T and four cases had mutations in exons 10, 11, or 15.

### *TERT* mRNA expression and telomerase activity in a subset of MTCs

**Figure 1.**
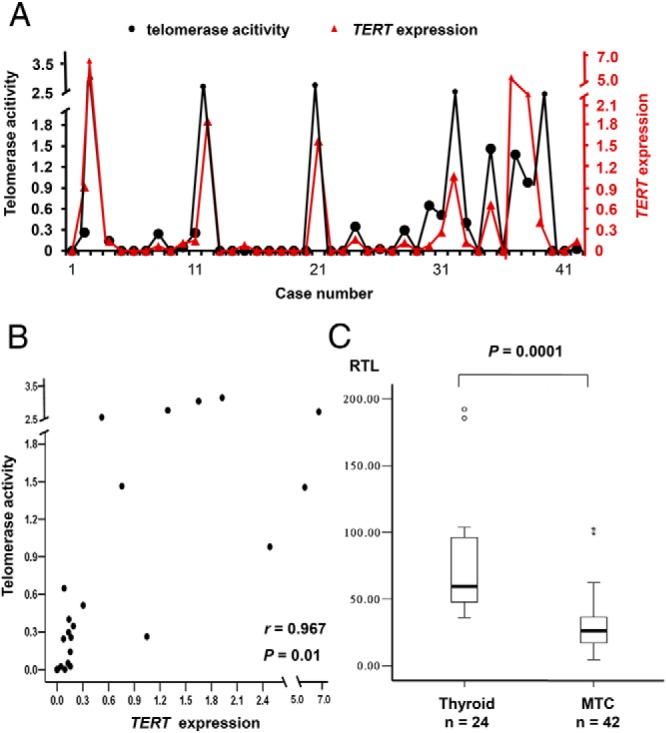
Correlation between *TERT* mRNA expression and telomerase activity. A) Comparison of relative telomerase activity (filled circle) and relative *TERT* gene expression (red triangle) for each of the 42 cases. The left y-axis suggests the telomerase activity and the right y-axis refers to the *TERT* expression values given in arbitrary units. B) Scatter diagram of relative *TERT* expression and relative telomerase activity in the 42 MTCs shows a strong positive correlation between two variables (*P* = .01, Spearman rank order correlation). C) Comparison of relative telomere length (RTL) given in arbitrary units in MTCs and thyroid tissues. Box plots show mean telomere lengths determined by real-time PCR. MTCs had significantly shorter telomeres as compared with thyroid tissue. The statistical analyses were performed using Mann-Whitney *U* test. Cases suggested with closed circle are outliers.

Among the 39 sporadic cases, 21 (54%) displayed *TERT* mRNA expression whereas 18 (46%) were negative (Figure 1A). The three MEN 2 cases did not reveal any *TERT* expression. Relative telomerase activity was then detected in the same 21 sporadic MTCs (54%), whereas the remaining 18 sporadic cases as well as three MEN 2 cases, the thyroid tissues, and the negative control were all negative. Although the relative telomerase activity showed variation between individuals (mean OD value, 1.04; range, 0.02–3.25), it showed strong positive correlation with the *TERT* gene expression (*r* = 0.967, *P* = .01) (Figure 1B).

### Reduced mean telomere length in MTC

In the MTCs the mean telomere lengths were significantly shorter compared with thyroid tissue samples (*P* = .0001; Figure 1C). Moreover, the relative telomere length of tumors with telomerase activation was shorter compared with the rest, although the difference was not significant (data not shown). However, the mean telomere length was not associated with clinical parameters of sporadic MTC cases (data not shown).

### Association between telomerase activation and clinical features of sporadic MTC

Sporadic MTCs with and without telomerase activation were compared for clinical phenotypes and follow-up. As shown in Table 1, telomerase activation was significantly more frequent in male compared with female patients (*P* = .02) and correlated with a more advanced stage at diagnosis (*P* < .0001) as well as larger tumors (*P* = .027). All telomerase-positive cases had stage III or stage IV disease. In the telomerase-negative group, nine patients had stage I or II disease and nine patients had stage III or IV disease. The three patients with MEN 2 had stage I or II disease. Furthermore, there was a positive correlation between telomerase activity and MIB-1 proliferation index (*r* = 0.683, *P* = .01; Supplemental Figure 1). These findings suggest that telomerase activation is associated with more aggressive growth in sporadic MTC.

**Table 1.** Comparison of Telomerase Activation with Characteristics of Sporadic MTC

Parameter	Telomerase Activation	Telomerase Negative	*P* Value^a^
Informative cases (n)	21	18	
Age at diagnosis (n = 39)			
Mean (min.-max.), y	53 (13–87)	58.2 (21–75)	.294
Sex (n = 39)			**.019**
Female	9	15	
Male	12	3	
Tissue type (n = 38)			.282
Primary tumor	13	14	
Metastasis	8	3	
Tumor size*^b^* (n = 38)			**.027**
T1	4	7	
T2	5	7	
T3	3	4	
T4	8	0	
TNM stage (n = 39)			<**.0001**
Stage I	0	3	
Stage II	0	6	
Stage III	1	5	
Stage IV	20	4	
MIB1 proliferation index (n = 22)			.134
0–2% positive nuclei	5	7	
3–10% positive nuclei	5	2	
>10% positive nuclei	3	0	
Follow-up (n = 39)			**.005**
Mean (min.-max.), y	7.3 (1–28)	13.7 (2–35)	
Outcome (n = 39)			<**.0001**
Alive and disease free	0	12	
Alive with spread disease	8	5	
Dead (resulting from disease)	13 (12)	1 (0)	

a Significant *P*-values are indicated in bold.

b T1, <20 mm; T2, 20–40 mm; T3, >40 mm but limited to thyroid; T4, tumor extends beyond the thyroid capsule.

### Telomerase activation as a prognostic factor of MTC patient survival

**Figure 2.**
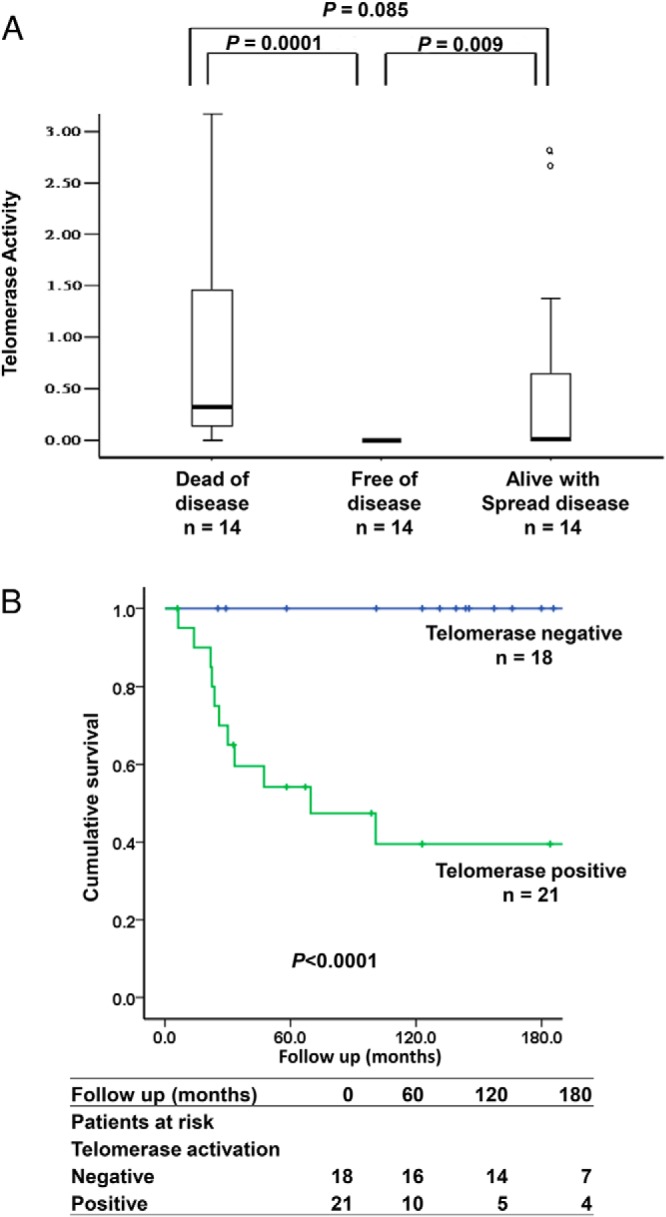
Telomerase is a prognostic marker for survival of sporadic MTC patients. A) Box plot shows relative telomerase activity in MTC tumors from patients with different clinical outcomes. Differences between groups were evaluated by Mann-Whitney *U* test. Cases labeled with circle are outliers. B) Kaplan-Meier plots showing significantly shorter survival for patients with telomerase activation as compared with cases without telomerase activation (*P* < .0001). The statistical analysis was performed using log-rank test.

Sporadic MTC cases were divided in two groups according to the outcomes at follow-up, including cases free of disease (alive and free of disease or dead resulting from other cause) and cases with persistent disease (alive with persistent disease or dead resulting from disease), and compared concerning clinical phenotypes and molecular features. Telomerase expression and telomerase activity were significantly associated with poor outcome (Table 2) as well as late-stage disease. Among the 21 telomerase-positive sporadic MTC cases, 12 died as a result of MTC, one died as a result of renal failure, and 8 were alive with spread disease at the end of follow-up. In the telomerase-negative group, all patients were alive except one who died as a result of lymphoma, including 12 who were free of disease and 5 patients with spread disease. Telomerase activity had a positive association with different outcomes; cases free of disease showed the lowest telomerase activity (Figure 2A). The Kaplan-Meier analysis showed a significantly shorter survival in the group with telomerase activation (*P* < .0001) (Figure 2B). Hence, we found a strong correlation between clinical outcome and telomerase activation (*P* < .0001). The multivariate analysis performed by logistic regression test showed that only telomerase activation was independently correlated with a poor outcome in MTC patients (odds ratio (OR), 24; 95% CI, 1.8–339; *P* = .017). Stage did not reach statistical significance in the multivariate analysis (OR, 3; 95% CI, 0.35–27; *P* = .345).

**Table 2.** Comparison of Telomerase and *TERT* Findings of Sporadic MTC with Final Outcome

Parameter	Free of Disease	Persistent Disease	*P* Value^a^
Informative cases (n)	14	25	
Telomerase activation (n = 39)			**.0001**
Postive	1	20	
Negative	13	5	
TERT mRNA expression (n = 39)			**.0001**
Postive	1	20	
Negative	13	5	
Telomerase activity (n = 39)			**.0001**
Mean	0.23	0.73	
Telomere length (n = 39)			.46
Mean (min.-max.)	0.86 (0.5–1.6)	0.83 (0.3–2.1)	
TERT promoter mutation (n = 39)*^b^*	0	0	

a Significant *P* values are indicated in bold.

b *TERT* promoter mutation data have been published in (15).

We found that telomerase activation was associated with aggressive disease and poor clinical outcome. Given that *RET* M918T is reported to be a marker of poor prognostic in MTC, we compared these two parameters in 39 sporadic MTC cases. However, no significant association was detected between M918T mutation and telomerase activation (Supplemental Figure 2).

### *TERT* alternative splice variants in MTCs

**Figure 3.**
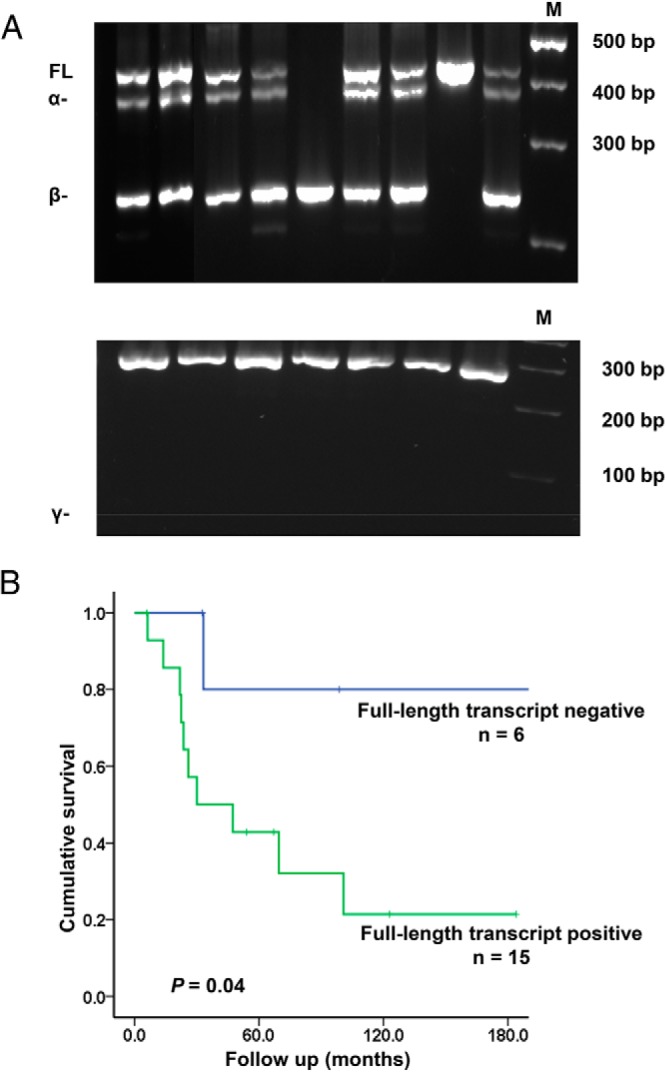
Detection of *TERT* alternative splice variants in MTC. A) Representative electrophoretic gels showing the *TERT* full-length variant (FL), the α deletion (α^−^) and the β deletion (β^−^) detected by nested PCR. As shown in the lower panel the γ deletion (γ^−^) was not detected in this study. B) Kaplan-Meier plots showing significantly shorter survival for patients with the telomerase full-length transcript as compared with cases without the full-length transcript (*P* = .04). The statistical analysis was performed using log-rank test.

*TERT* expression was further characterized concerning alternative splice variants in the 21 sporadic cases with positive expression of total *TERT*. Four *TERT* alternative splice variants were determined. Three of these four were detected in subsets of the tumors, as illustrated for representative cases in Figure 3A. Fourteen of the 21 cases exhibited a combination of full-length transcript, inhibitory α^−^ deletion (382 bp), nonfunctional β^−^ deletion (236 bp), and one case only showed full-length transcript. The remaining six cases showed the nonfunctional β^−^ deletion only. The γ^−^ deletion (91 bp) was not detected in any of the 21 cases examined. Cases with full-length transcript tended to have higher telomerase activity and shorter relative telomere length; however, the differences were not statistically significant (data not shown). Survival analysis showed a significantly shorter survival in the group with full-length transcript compared with cases without full-length transcript (*P* = .04) (Figure 3B). Hence, we found a correlation between clinical outcome and *TERT* full-length transcript. However, the presence or absence of full-length or α^−^ deletion transcript was neither associated with other clinical parameters nor with *RET* M918T mutation. Seven of the 15 cases expressing the full-length transcript showed high levels of full-length *TERT* (>35% of the total *TERT*); however, there was no difference concerning clinical parameters for cases with high or low expression levels of full-length *TERT*.

### Detection of the ALT activation in telomerase-negative MTCs

**Figure 4.**
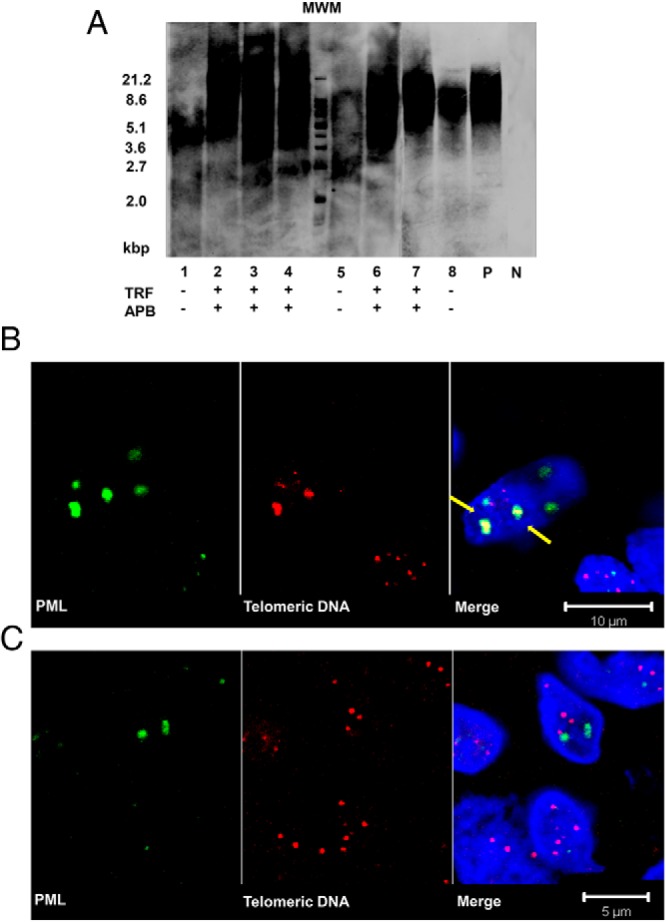
Detection of ALT in telomerase-negative MTC. A) Southern blot analysis of terminal restriction fragment (TRF) shows the distribution of telomere length in telomerase-negative MTC. The length of telomeres of sample 2, 3, 4, 6, and 7 ranged from <2 kb to >21.2 kb. The length of telomeres of sample 1, 5, and 8 ranged from 3–8 kb, which had a more homogeneous distribution and a shorter telomere length. The heterogeneous distribution was scored as TRF (+), and detection of APBs is suggested below. Molecular weight marker (MWM), and positive (P) and negative (N) controls are included. B and C) Combined PML immunofluorescence (green) and telomere FISH (red). B) Analysis of MTC cases at ×100 magnification. Arrows suggest PML colocalized with telomere DNA foci, representing APBs. C) Tel-FISH performed in normal noncancerous thyroid control tissue.

To explore whether the ALT mechanism is involved in MTC cases without telomerase activation, we performed Southern blot analysis of 17 telomerase-negative and 6 telomerase-positive MTCs to evaluate the length of telomeres. As exemplified in Figure 4A, 11 of 17 telomerase-negative MTC cases (65%) exhibited a heterogeneous distribution of telomere lengths that is the characteristic of ALT-positive cells whereas telomerase-positive cases displayed a homogeneous pattern. Tel-FISH was performed on embedded tissue from nine telomerase-negative samples, 10 telomerase-positive samples, and five thyroid tissues. The prevalence of APBs varied among the cases. Six of nine telomerase-negative slides (67%) showed APBs whereas the rest had undetectable APB (Figure 4). As expected, APBs were not found in five thyroid and 10 telomerase-positive MTC tissues. The observed telomere length heterogeneity and APBs suggest that the ALT mechanism is involved in a subset of sporadic MTC. Intriguingly, several tumors showed neither telomerase activation nor involvement of ALT. ALT-positive tumors showed lower MIB-1 proliferation index (median, 1%; range, 1–3%) compared with ALT-negative cases (median, 3%; range, 1–80%) (*P* = .024; Supplemental Figure 1).

## Discussion

In the present study we found activation of telomerase in approximately half the MTC cases. Three different splice isoforms of *TERT* were detected among the telomerase positive cases; cases with expression of full-length transcripts showed a shorter survival. Telomerase activation had strong prognostic influence on patient survival but was independent of stage and the common *RET* M918T mutation. Activation of the ALT mechanism was demonstrated while a subset of cases was negative for both telomerase and ALT, suggesting that additional mechanisms of telomere stabilization might be operative in MTC.

Telomerase is a multienzymatic complex, which can be regulated at different levels (25). Because *TERT* gene expression is not always correlated with the enzyme activity, many studies have found that either the telomerase activity or the *TERT* expression is a marker of tumor aggressiveness and poor prognosis. In our study, there was a significant concordance between telomerase activity and *TERT* expression (*r* = 0.9). Still, some MTCs showed high *TERT* expression together with low telomerase activity. This observation could be related to differences in post-transcriptional modifications, translational efficiency, expression of other telomerase components, or TERT phosphorylation. We found that half of the investigated MTCs showed telomerase activation. Taken together, previously published studies have also reported recurrent telomerase-positive and -negative MTCs. However, because most studies are based on one or a few MTC cases only, direct comparisons of frequencies is not meaningful (Supplemental Table 3). Furthermore, the result may vary depending on the cases included concerning sporadic or MEN 2–related MTC as well as metastatic or nonmetastatic cases. Interestingly, low levels of telomerase activity were reported in MTCs compared with HEK-293 cells (26), and even low telomerase activity of less than 5% of HEK-293 may prolong the replicative life span of MTC cell strains (27).

Approximately two thirds of the patients in the telomerase-positive group died as a result of MTC and the remaining patients had spread disease. Telomerase activation was independently correlated with a worse outcome of MTC; cases with telomerase activation had a significantly shorter survival. In adults, telomerase is silent in most tissues whereas expressed in stem cells, male germ line cells, and activated immune cells only. Somatic cells with short telomeres enter a senescent or apoptosis state after several divisions without activating telomerase (28, 29). However, some cells could bypass the checkpoints and avoid entering crisis by activating a mechanism of telomere maintenance. The primary mechanism for this is activation of telomerase. As shown in this study, half of the MTC cases used the primary mechanism to stabilize telomeres, i.e. telomerase activation. The role of telomerase is generally regarded as maintaining telomere length. However, accumulating evidence suggests that telomerase have additional functions in regulation of cell growth and survival, which may contribute to tumorigenesis (30). Here, although telomerase was not universally activated in MTCs, the frequency of telomerase activation was significantly associated with poor outcome, with large and late-stage tumors, demonstrating that up-regulation of telomerase is a relatively important event in MTC development. Several clinical and histopathological parameters such as lymph node invasion and distant metastases are important prognostic factors. Therefore, the association between telomerase activation and aggressive behavior (poor clinical outcome and advanced tumor stage), could be used to predict the prognosis of MTC patients, aiding in decision making for follow-up and additional therapy.

The *TERT* gene is known to generate seven alternative splice forms and produce multiple transcripts; here we detected three *TERT* splice forms. The α^−^ deletion transcript has a reverse transcriptase motif A and can negatively regulate telomerase activity, whereas the β^−^ deletion and the γ^−^ deletion transcripts are without any known function (17, 20, 21). In a previous study of other cancers, telomerase activity was shown to depend on the existence of full-length *TERT* gene expression (18, 19). We found that most of the MTCs with full-length transcript exhibited relatively high telomerase activity. However, a few other cases exhibited high telomerase activity with low expression of the full-length splice form. Hence, in MTC telomerase, activity was not fully dependent of expression of the full-length transcript. The cases with full-length transcripts showed a shorter survival, implying that full-length *TERT* play an important role in telomerase functions in MTC.

Telomerase activation is the predominant mechanism of telomere maintenance in most malignancies. However, the mechanism for activation of telomerase in vivo is not fully understood. Some type of genetic event is suggested such as gene amplification, duplication, and translocation of the *TERT* locus, or the activating *TERT* promoter mutation. In our previous study, *TERT* promoter mutation led to telomerase activation in papillary and follicular thyroid cancer (15). However mutations were not revealed in the same series of MTCs investigated here (15), demonstrating that the telomerase activation was not a result of *TERT* promoter mutation. We also investigated the possible connection between telomerase activation and the common M918T *RET* mutation, which is associated with a less favorable clinical outcome (10, 31). In the present study, this M918T mutation was not significantly associated with specific clinical characteristics. Telomerase activation was twice as frequently observed in the M918T mutation group than the group without M918T mutation. However, the difference did not reach statistical significance (*P* = .065). Telomerase may be proliferation regulated, and there is a positive correlation between *RET* mutations and Ki-67 expression in MTC (32). In the present study, MIB-1 proliferation index was positively correlated with telomerase activity and negatively associated with ALT involvement.

Besides the primary mechanism of telomere stabilization by telomerase activation, ALT-based chromosome recombination is involved in a subset of cancers (7, 33). We found involvement of ALT in a subset of MTC cases without telomerase activation. Heterogeneity in telomere length was observed in 11 of 17 cases, and presence of APBs was observed in six telomerase-negative cases. This is in accordance with previous observations of features of ALT, and it is the first description of a telomerase-independent telomere mechanism in MTC. The ALT mechanism has been described for tumor cells lacking telomerase activation (34), which is common in sarcomas and glioblastoma multiforme. The ALT activation is thought to be based on homologous recombination and copy switching of telomeric repeats. Telomere shortening in the absence of telomerase followed by recombination results in heterogeneous telomere length and the APBs are storage places for DNA synthesis (35). A subset of telomerase-negative MTCs exhibited heterogeneous telomeres and APBs, which was in accordance with the results of chromosomal instability. All these findings suggest the existence of the ALT mechanism in MTCs.

Of interest is the observation that some MTC cases exhibited neither telomerase activation nor features of ALT, which may suggest the existence of additional effective mechanisms of telomere stabilization. Two groups described an SV40-immortalized human cell line with neither telomerase activation nor APBs (36, 37), and another group showed the presence of an efficient telomere stabilization mechanism different from telomerase activation and ALT in non–small-cell lung cancer cell lines (38). In addition, a significant number of cancer cell lines maintain telomeres without signs of telomerase activation or ALT (39). These studies support the existence of other telomere stabilization mechanisms besides telomerase and ALT activation, which is in agreement with the present study.

The telomere length is the result of a dynamic balance between shortening and elongation. Short telomeres are associated with poor prognosis of human carcinomas including thyroid cancer (40). The presence of short telomeres has been reported in patients with sporadic cancers of for example bladder, lung, kidney, and head and neck (4). We found that MTCs presented shorter telomeres compared with thyroid tissues, which is in accordance with previous observations in other cancers.

In conclusion, at least two mechanisms are involved in telomere maintenance in MTC including telomerase and ALT activation. Alternative splicing of *TERT* partly accounts for activation of the telomerase protein. Telomerase activation is of prognostic importance in MTC, and could serve as a prognostic marker for MTC patients. In addition, our data imply the existence of other possible mechanisms for telomere stabilization in MTC. Hence, MTC might be a useful tool for investigating the molecular background of telomere stabilization.
